# Raised D-dimer Level among COVID-19 Patients in a Tertiary Care Hospital: A Descriptive Cross-sectional Study

**DOI:** 10.31729/jnma.7311

**Published:** 2022-03-31

**Authors:** Shikha Rizal, Bishal Raj Joshi, Sunil Regmi

**Affiliations:** 1 Department of Biochemistry, Nobel Medical College Teaching Hospital, Biratnagar-4, Nepal; 2 Department of Urology, Koshi Hospital, Biratnagar-9, Nepal

**Keywords:** *corona virus disease*, *d-dimer*, *disease severity*

## Abstract

**Introduction:**

Serum D-dimer is a protein fragment generated during the final phase of clot formation. Increased serum D-dimer levels indicate the hemostatic change in patients, likely related to the prothrombotic switch. As the world is battling with the damaging effect of coronavirus disease, it is very important to find out the early and effective predictors of prognosis to improve the management of COVID-19 patients. Thus, our study aims to find out the prevalence of increased D-dimer levels in coronavirus disease patients.

**Methods:**

A descriptive cross-sectional study was conducted on a total of 235 patients admitted in the COVID ward and COVID Intensive Care Units at a tertiary care hospital from July 2020 to August 2021 after getting ethical approval (Reference number: 401/2020) from the Institutional Review Committee. A convenience sampling method was used for sample collection. The highest recorded values for D-dimer during the hospital stay were taken for data collection. The data were entered in Microsoft Excel 2013 and analyzed using Statistical Package for the Social Sciences version 16.0. Point estimate at 95% Confidence Interval was calculated along with frequency, proportion, mean and standard deviation.

**Results:**

Among 235 patients, elevated D-dimer level was in 175 (74.46%) (68.88-80.04 at 95% Confidence Interval). Majority of the patients were males 136 (77.71%) whereas 39 (22.28%) of the patients were females.

**Conclusions:**

The prevalence of raised D-dimer levels was quite higher in our studies compared to other studies done in different parts of the world. Thus, serum D-dimer level may serve as an early marker in improving the management of patients with coronavirus disease.

## INTRODUCTION

The world continues to battle the damaging effect of coronavirus disease (COVID-19), which has become a public health emergency since December 2019.^1,2^ Besides the respiratory manifestations of COVID-19, a major complication happens to be the consistent hemostatic changes in patients with severe disease, likely related to the pro-thrombotic switch.^2^ Presence of thrombi within the pulmonary vasculature is associated with a diffuse alveolar change in most patients who died from the disease.^3^ Thus, early and effective predictors of clinical outcome is necessary to improve the management of COVID-19 patients.^4^

D-dimer is the main product of degradation of crosslinked fibrin by plasmin which is generated in the final phase of clot formation.^5^ It is normally undetectable or detectable at a very low level in the blood, but its level rises in the blood when there is the activation of coagulation and fibrinolysis.^5,6^ Thus, D-dimer being a sensitive marker of thrombosis, it is currently the best available marker for hemostatic abnormalities.

Our study aimed to determine the prevalence of increased serum levels of D-dimer in patients with COVID-19.

## METHODS

A descriptive cross-sectional study was conducted on a total of 235 patients who were admitted to the COVID ward and COVID ICU at Nobel Medical College Teaching Hospital. This study was conducted from July 2020 to December 2020 and from March 2021 to August 2021 during the peak of infection in Nepal. Ethical approval was taken from the Institutional Review Committee (IRC) of the institution (Reference number: 401/2020) and informed consent was taken from each patient following the safety precaution guidelines for COVID-19.

Inclusion criteria for selection of patients were: Adult patients aged >18 years who were diagnosed as a positive result of real-time PCR assay of nasal and oropharyngeal swab and the patient must be clinically proven COVID pneumonia.

Exclusion criteria include patients with a known history of hematological abnormalities. A convenient sampling method was used for sample collection.

The sample size was calculated using the following formula:

n = (Z^2^ × p × q) / e^2^

  = (1.96^2^ × 0.5 × 0.5) / 0.07^2^

  = 196

Where,

n = minimum sample size required
Z = 1.96 at 95% Confidence Interval (CI)
p = prevalence taken as 50% for maximum sample size
q = 1-p
e = margin of error, 7%

Approximately 3 ml of venous blood was collected in a yellow capped plain vial from an antecubital vein under strict aseptic condition following the protocol for COVID-19. Centrifugation was done at 3000 rpm for 10 minutes and separated serum was analyzed for D-dimer. D-dimer was estimated by the immuno-turbidimetric method.

The normal value for d-dimer was taken as <0.5 mg/l according to the hospital's reference laboratory. The highest recorded values for D-dimer during the hospital were taken for data collection.

The data were entered in Microsoft Excel 2013 and analyzed using Statistical Package for the Social Sciences version 16.0. Point estimate at 95% Confidence Interval was calculated along with frequency and proportion for binary data, and mean and standard deviation for continous data.

## RESULTS

**Figure 1:**
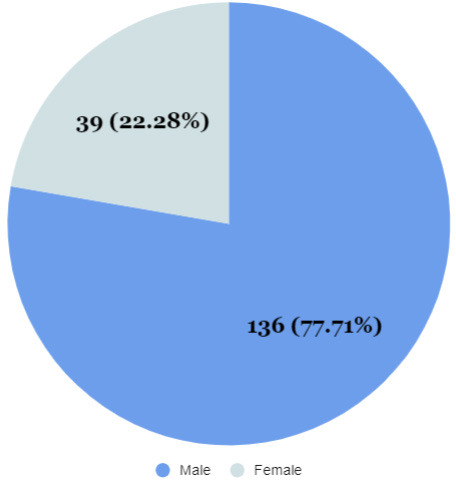
Gender distribution of COVID cases (n= 175).

Among 235 patients recruited for the study, the prevalence of elevated D-dimer level was 175 (74.46%) (68.88-80.04 at 95% Confidence Interval) with the D-dimer levels elevated in 39 (22.28%) of the females and 136 (77.71 %) of the males (Figure 1).

**Figure 2:**
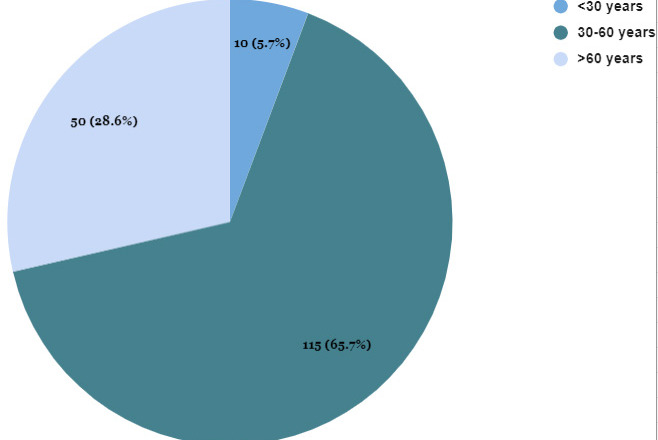
Age-wise distribution of the patients with elevated D-dimer levels (n= 175).

Among 175 patients who had elevated D-dimer levels, the age group 30-65 years comprised the majority in 115 (65.71%) (Figure 2).

The mean age of the study population was 52±15 years for males and 51±16 years for females. The median D-dimer level in males and females is expressed below (Table 1).

**Table 1:** Descriptive statistics of COVID patients according to gender distribution (n= 175).

	Male	Female
Age (Mean±SD)	52±15 years	51±16 years
D-dimer: Median (Interquartile Range)	1.18 mg/l (0.50-2.09)	0.96 mg/l (0.53-1.94)

The median value of D-dimer was found to be highest in patients with age groups above 60 years, as the median values were above the normal in all the age groups (Table 2).

**Table 2:** The median value of D-dimer levels according to age groups (n=175).

Age (years)	n (%)	D-dimer (mg/l) Median (Interquartile range)
0-30	10 (5.71)	1.87 (0.60-1.88)
30-60	115 (65.71)	2.32 (0.61-2.89)
>60	50 (28.57)	2.53 (0.60-3.39)

## DISCUSSION

On 30^th^ January 2020, WHO declared the outbreak of COVID-19 infection as a public health emergency of international concern.^7^ Since then a very high volume of patients are presenting to the hospital with the severe condition and we are still living with the threat of this viral infection expecting its third wave soon in the future.^4^ Thus, risk stratification measures along with early diagnosis would clearly be helpful. It has been reported that COVID-19 is associated with hemostatic abnormalities,^4^ hence a measurement of serum D-dimer levels can be very useful.

Out of 235 patient recruited in our study, increased levels of D-dimer was found to be in 175 (74.46%) of the COVID patients. The majority of the patients in our study with raised D-dimer were males representing 139 (78%), whereas only 39 (22%) were females. Our findings were similar to the study done by Khatri P, et al. in western Nepal, where males represented 62% of their total sample size.^3^

The mean age of our study participants was 52±15 years for males and 51±16 for females respectively. These findings were consistent with the studies done by Wright FL, et al, Cui S, et al. where the mean age was 51.7 years and 59.9 years respectively, clearly stating the fact that most of the infected patients requiring hospitalization were of the age group 50-60 years.^8,9^ Also when we analyzed the age distribution of the COVID cases, the majority of our patients were of the age group 30-60 years which represents 115 (66%) of our sample size.

Several meta-analyses have been done to compare the level of serum D-dimer and have proved the ability of laboratory tests to assess the severity of the disease.^2,10,11^ and also the laboratory biomarkers altered in COVID-19 has guided the clinicians to highlight the pathological mechanism of the disease.^12^

D-dimer is an indirect marker of active coagulation and thrombus formation, which is released when plasmin, a fibrinolytic enzyme cleaves fibrin to degrade clots and represent a mirror of endovascular thrombotic processes.^11^ In our study, the median D-dimer level was 1.18 mg/l (Interquartile range 0.50-2.09) in males and 0.96 mg/l (Interquartile range 0.53-1.94) in females, which was significantly higher in both the sexes when compared with the normal levels of d-dimer (>0.5 mg/l). This finding of our study was consistent with the studies done by Rostami M, et al. where the median serum D-dimerlevels were 1.53 mg/l.^13^ Genderwise distribution of D-dimer levels represents, elevated D-dimer level in almost 136 (77.71%) of the males and 39 (22.28%) of the females. This finding was similar to the study done by Xiaokang He, et al. where the D-dimer level was found to be elevated in almost 54.4% of the cases. Also, when we see the age-wise distribution of the D-dimer levels, it was highest in the age group above 60 years which was 2.53 mg/l. A similar finding was seen in a study done by Xiaokang He, et al. which reported that most of the patients with elevated D-dimer levels were above 60 years of age.^14^ Recent studies have reported that D-dimer levels were significantly associated with severity of COVID,^15^ hence a significant correlation between high levels of D-dimer and survival rates underlines the importance of detection of D-dimer levels in COVID-19 patients. D-dimer, thus is a commonly tested parameter in hospitalized patients with COVID-19.

The rise in the D-dimer level reflects the degradation product of fibrin accumulating in the alveoli, coming to the blood. Thus, it is understood that D-dimer is associated with the degree and prognosis of lung injury.^16^

This was a single-centre study with a small sample size so cannot be generalized for all. Further assessment of the discharged patients and their laboratory tests were not recorded.

## CONCLUSIONS

Thus, the prevalence of increased D-dimer was high in our hospital compared to other similar studies. Thus, this easy and inexpensive laboratory test can be used as a biomarker to assess disease severity and clinical outcome. Also, this biomarker may help clinicians to quickly triage patients for risk stratification and timely management.
